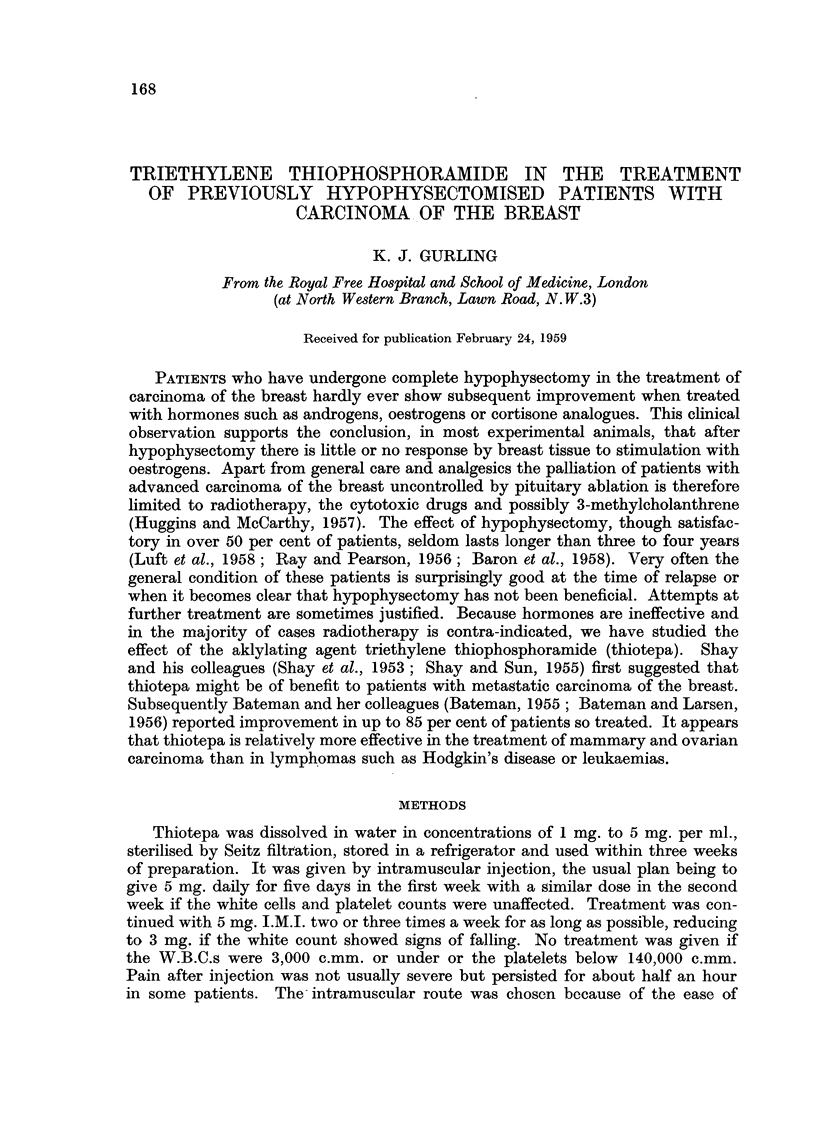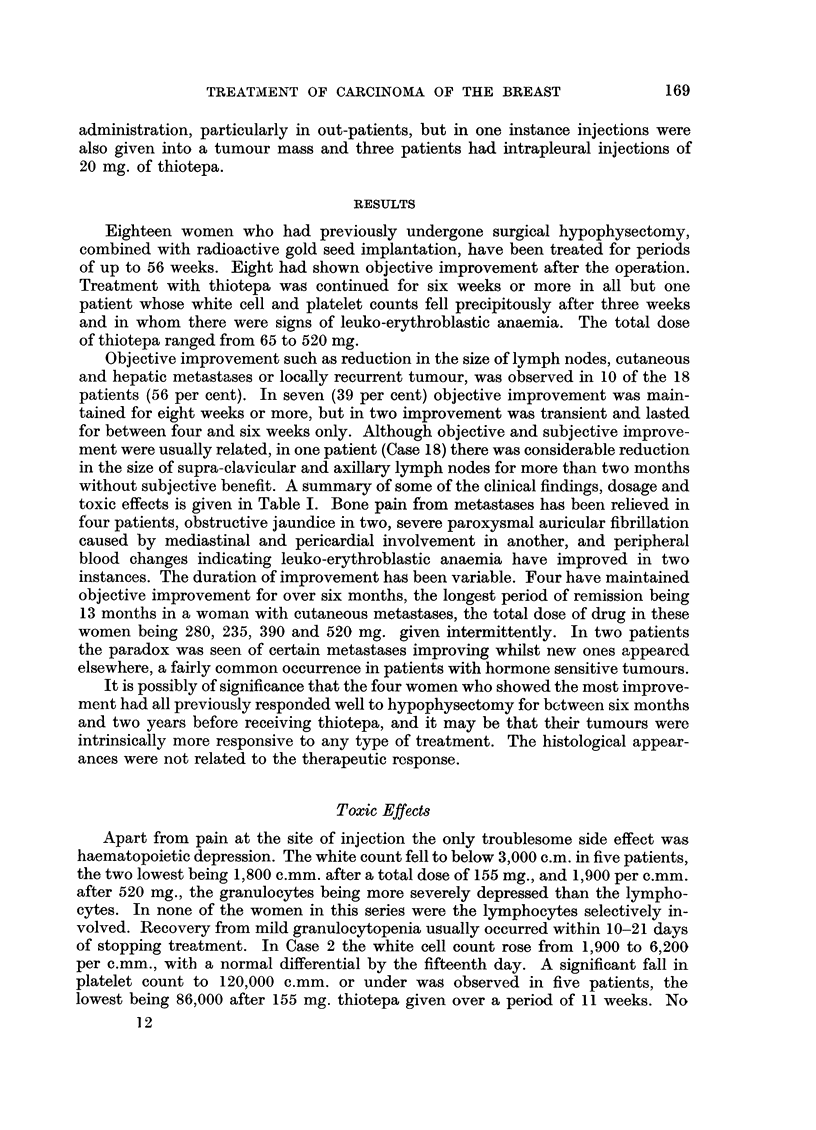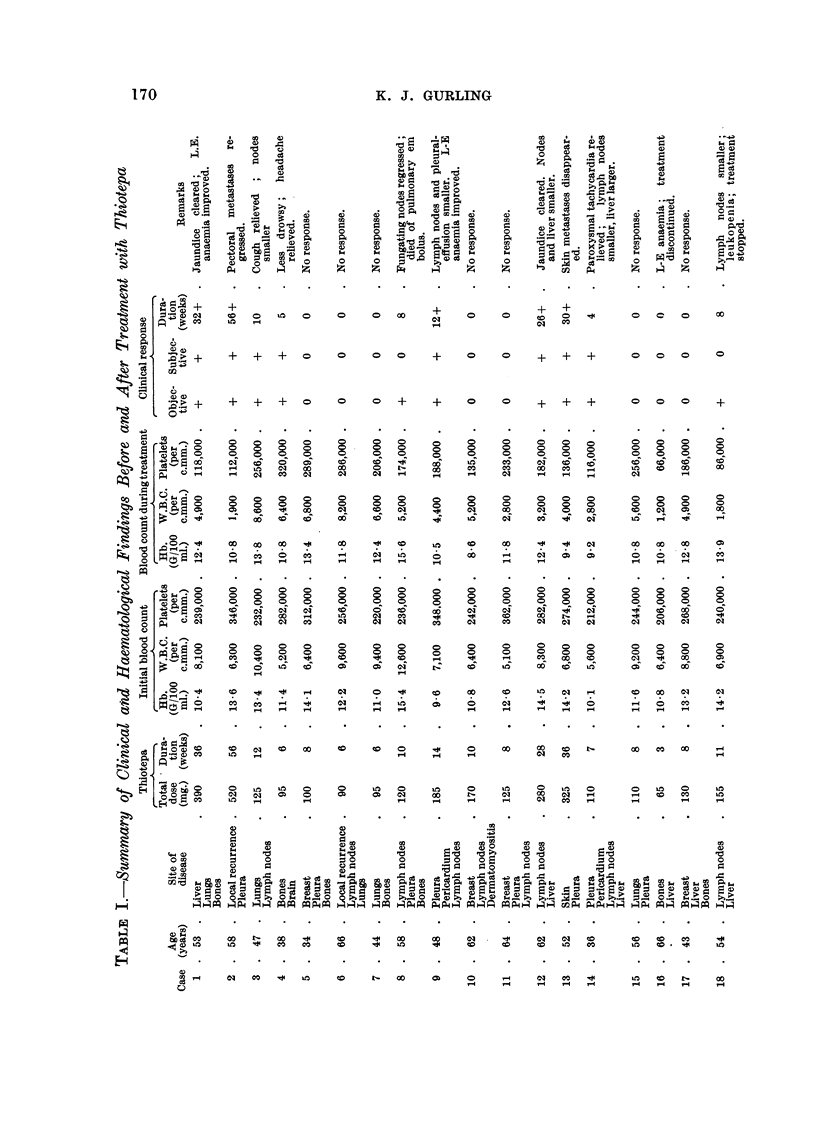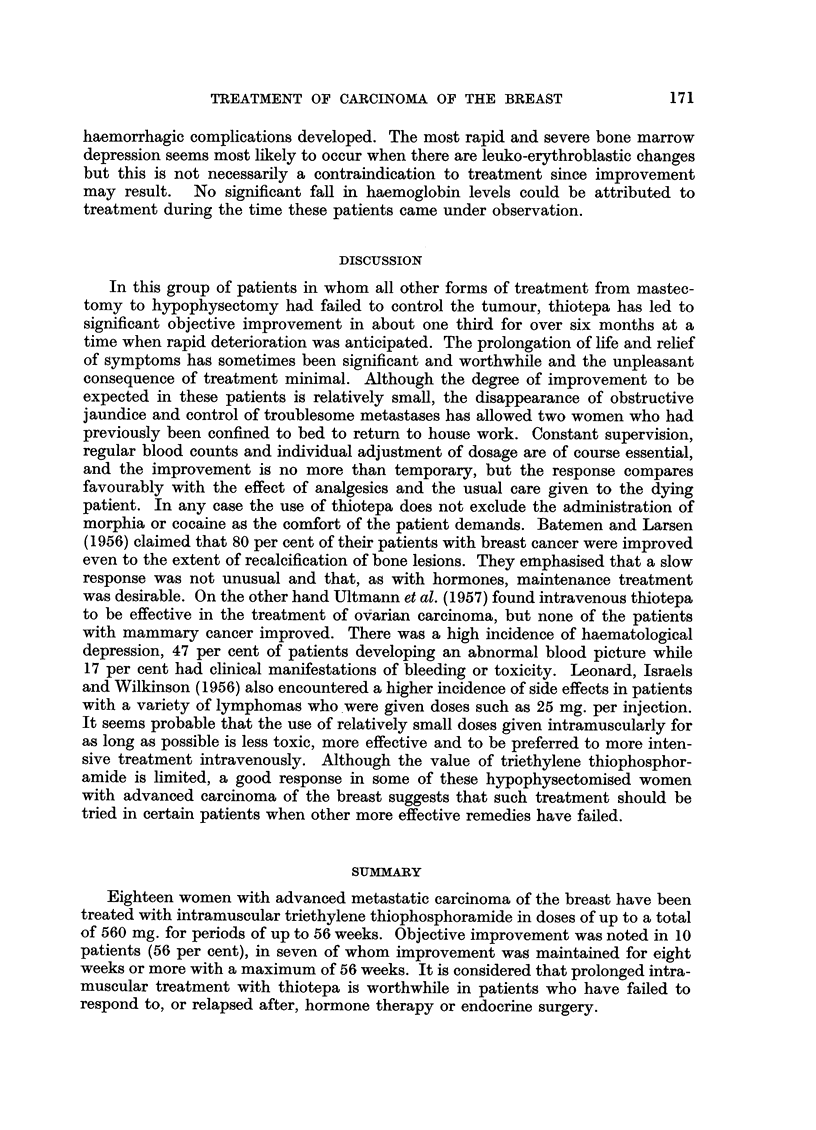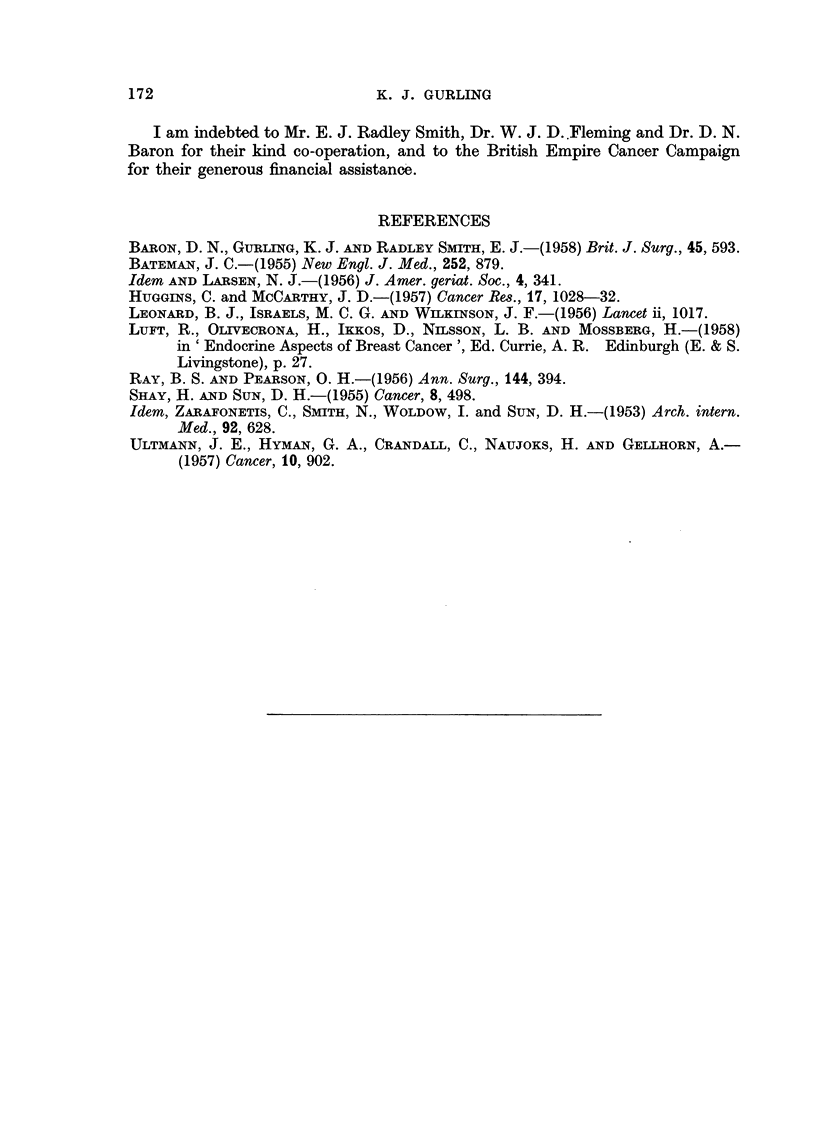# Triethylene Thiophosphoramide in the Treatment of Previously Hypophysectomised Patients with Carcinoma of the Breast

**DOI:** 10.1038/bjc.1959.23

**Published:** 1959-06

**Authors:** K. J. Gurling


### TRIETHYLENE THIOPHOSPHORAMIDE IN THE TREATMENT OF PREVIOUSLY HYPOPHYSECTOMISED PATIENTS WITH CARCINOMA OF THE BREAST

K. J. GURLING

*From the Royal Free Hospital and School of Medicine, London (at North Western Branch, Lawn Road, N.W.3)*

Received for publication February 24, 1959

PATIENTS who have undergone complete hypophysectomy in the treatment of carcinoma of the breast hardly ever show subsequent improvement when treated with hormones such as androgens, oestrogens or cortisone analogues. This clinical observation supports the conclusion, in most experimental animals, that after hypophysectomy there is little or no response by breast tissue to stimulation with oestrogens. Apart from general care and analgesics the palliation of patients with advanced carcinoma of the breast uncontrolled by pituitary ablation is therefore limited to radiotherapy, the cytotoxic drugs and possibly 3-methylcholanthrene (Huggins and McCarthy, 1957). The effect of hypophysectomy, though satisfactory in over 50 per cent of patients, seldom lasts longer than three to four years (Luft *et al.*, 1958 ; Ray and Pearson, 1956 ; Baron *et al.*, 1958). Very often the general condition of these patients is surprisingly good at the time of relapse or when it becomes clear that hypophysectomy has not been beneficial. Attempts at further treatment are sometimes justified. Because hormones are ineffective and in the majority of cases radiotherapy is contra-indicated, we have studied the effect of the aklylating agent triethylene thiophosphoramide (thiotepa). Shay and his colleagues (Shay *et al.*, 1953 ; Shay and Sun, 1955) first suggested that thiotepa might be of benefit to patients with metastatic carcinoma of the breast. Subsequently Bateman and her colleagues (Bateman, 1955 ; Bateman and Larsen, 1956) reported improvement in up to 85 per cent of patients so treated. It appears that thiotepa is relatively more effective in the treatment of mammary and ovarian carcinoma than in lymphomas such as Hodgkin's disease or leukaemias.

### METHODS

Thiotepa was dissolved in water in concentrations of 1 mg. to 5 mg. per ml., sterilised by Seitz filtration, stored in a refrigerator and used within three weeks of preparation. It was given by intramuscular injection, the usual plan being to give 5 mg. daily for five days in the first week with a similar dose in the second week if the white cells and platelet counts were unaffected. Treatment was continued with 5 mg. I.M.I. two or three times a week for as long as possible, reducing to 3 mg. if the white count showed signs of falling. No treatment was given if the W.B.C.s were 3,000 c.mm. or under or the platelets below 140,000 c.mm. Pain after injection was not usually severe but persisted for about half an hour in some patients. The intramuscular route was chosen because of the ease of administration, particularly in out-patients, but in one instance injections were also given into a tumour mass and three patients had intrapleural injections of 20 mg. of thiotepa.

### RESULTS

Eighteen women who had previously undergone surgical hypophysectomy, combined with radioactive gold seed implantation, have been treated for periods of up to 56 weeks. Eight had shown objective improvement after the operation. Treatment with thiotepa was continued for six weeks or more in all but one patient whose white cell and platelet counts fell precipitously after three weeks and in whom there were signs of leuko-erythroblastic anaemia. The total dose of thiotepa ranged from 65 to 520 mg.

Objective improvement such as reduction in the size of lymph nodes, cutaneous and hepatic metastases or locally recurrent tumour, was observed in 10 of the 18 patients (56 per cent). In seven (39 per cent) objective improvement was maintained for eight weeks or more, but in two improvement was transient and lasted for between four and six weeks only. Although objective and subjective improvement were usually related, in one patient (Case 18) there was considerable reduction in the size of supra-clavicular and axillary lymph nodes for more than two months without subjective benefit. A summary of some of the clinical findings, dosage and toxic effects is given in Table I. Bone pain from metastases has been relieved in four patients, obstructive jaundice in two, severe paroxysmal auricular fibrillation caused by mediastinal and pericardial involvement in another, and peripheral blood changes indicating leuko-erythroblastic anaemia have improved in two instances. The duration of improvement has been variable. Four have maintained objective improvement for over six months, the longest period of remission being 13 months in a woman with cutaneous metastases, the total dose of drug in these women being 280, 235, 390 and 520 mg. given intermittently. In two patients the paradox was seen of certain metastases improving whilst new ones appeared elsewhere, a fairly common occurrence in patients with hormone sensitive tumours.

It is possibly of significance that the four women who showed the most improvement had all previously responded well to hypophysectomy for between six months and two years before receiving thiotepa, and it may be that their tumours were intrinsically more responsive to any type of treatment. The histological appearances were not related to the therapeutic response.

### *Toxic Effects*

Apart from pain at the site of injection the only troublesome side effect was haematopoietic depression. The white count fell to below 3,000 c.m. in five patients, the two lowest being 1,800 c.mm. after a total dose of 155 mg., and 1,900 per c.mm. after 520 mg., the granulocytes being more severely depressed than the lymphocytes. In none of the women in this series were the lymphocytes selectively involved. Recovery from mild granulocytopenia usually occurred within 10–21 days of stopping treatment. In Case 2 the white cell count rose from 1,900 to 6,200 per c.mm., with a normal differential by the fifteenth day. A significant fall in platelet count to 120,000 c.mm. or under was observed in five patients, the lowest being 86,000 after 155 mg. thiotepa given over a period of 11 weeks. No

TABLE I.—*Summary of Clinical and Haematological Findings Before and After Treatment with Thiotepa*

| Case | Age (years) | Site of disease | Thiotepa: Total dose (mg.) | Thiotepa: Duration (weeks) | Initial blood count: Hb. (G/100 ml.) | Initial blood count: W.B.C. (per c.mm.) | Initial blood count: Platelets (per c.mm.) | Blood count during treatment: Hb. (G/100 ml.) | Blood count during treatment: W.B.C. (per c.mm.) | Blood count during treatment: Platelets (per c.mm.) | Clinical response: Objective | Clinical response: Subjective | Clinical response: Duration (weeks) | Remarks |
|---|---|---|---|---|---|---|---|---|---|---|---|---|---|---|
| 1 | 53 | Liver<br>Lungs<br>Bones | 390 | 36 | 10·4 | 8,100 | 239,000 | 12·4 | 4,900 | 118,000 | + | + | 32+ | Jaundice cleared; L.E. anaemia improved. |
| 2 | 58 | Local recurrence<br>Pleura | 520 | 56 | 13·6 | 6,300 | 346,000 | 10·8 | 1,900 | 112,000 | + | + | 56+ | Pectoral metastases regressed. |
| 3 | 47 | Lungs<br>Lymph nodes | 125 | 12 | 13·4 | 10,400 | 232,000 | 13·8 | 8,600 | 256,000 | + | + | 10 | Cough relieved; nodes smaller |
| 4 | 38 | Bones<br>Brain | 95 | 6 | 11·4 | 5,200 | 282,000 | 10·8 | 6,400 | 320,000 | + | + | 5 | Less drowsy; headache relieved. |
| 5 | 34 | Breast<br>Pleura<br>Bones | 100 | 8 | 14·1 | 6,400 | 312,000 | 13·4 | 6,800 | 289,000 | 0 | 0 | 0 | No response. |
| 6 | 66 | Local recurrence<br>Lymph nodes<br>Lungs | 90 | 6 | 12·2 | 9,600 | 256,000 | 11·8 | 8,200 | 286,000 | 0 | 0 | 0 | No response. |
| 7 | 44 | Lungs<br>Bones | 95 | 6 | 11·0 | 9,400 | 220,000 | 12·4 | 6,600 | 206,000 | 0 | 0 | 0 | No response. |
| 8 | 58 | Lymph nodes<br>Pleura<br>Bones | 120 | 10 | 15·4 | 12,600 | 236,000 | 15·6 | 5,200 | 174,000 | + | 0 | 8 | Fungating nodes regressed; died of pulmonary embolus. |
| 9 | 48 | Pleura<br>Pericardium<br>Lymph nodes | 185 | 14 | 9·6 | 7,100 | 348,000 | 10·5 | 4,400 | 188,000 | + | + | 12+ | Lymph nodes and pleural-effusion smaller. L-E anaemia improved. |
| 10 | 62 | Breast<br>Lymph nodes<br>Dermatomyositis | 170 | 10 | 10·8 | 6,400 | 242,000 | 8·6 | 5,200 | 135,000 | 0 | 0 | 0 | No response. |
| 11 | 64 | Breast<br>Pleura<br>Lymph nodes | 125 | 8 | 12·6 | 5,100 | 362,000 | 11·8 | 2,800 | 233,000 | 0 | 0 | 0 | No response. |
| 12 | 62 | Lymph nodes<br>Liver | 280 | 28 | 14·5 | 8,300 | 282,000 | 12·4 | 3,200 | 182,000 | + | + | 26+ | Jaundice cleared. Nodes and liver smaller. |
| 13 | 52 | Skin<br>Pleura | 325 | 36 | 14·2 | 6,800 | 274,000 | 9·4 | 4,000 | 136,000 | + | + | 30+ | Skin metastases disappeared. |
| 14 | 36 | Pleura<br>Pericardium<br>Lymph nodes<br>Liver | 110 | 7 | 10·1 | 5,600 | 212,000 | 9·2 | 2,800 | 116,000 | + | + | 4 | Paroxysmal tachycardia relieved; lymph nodes smaller, liver larger. |
| 15 | 56 | Lungs<br>Pleura | 110 | 8 | 11·6 | 9,200 | 244,000 | 10·8 | 5,600 | 256,000 | 0 | 0 | 0 | No response. |
| 16 | 66 | Bones<br>Liver | 65 | 3 | 10·8 | 6,400 | 206,000 | 10·8 | 1,200 | 66,000 | 0 | 0 | 0 | L-E anaemia; treatment discontinued. |
| 17 | 43 | Breast<br>Liver<br>Bones | 130 | 8 | 13·2 | 8,800 | 268,000 | 12·8 | 4,900 | 186,000 | 0 | 0 | 0 | No response. |
| 18 | 54 | Lymph nodes<br>Liver | 155 | 11 | 14·2 | 6,900 | 240,000 | 13·9 | 1,800 | 86,000 | + | 0 | 8 | Lymph nodes smaller; leukopenia; treatment stopped. |

haemorrhagic complications developed. The most rapid and severe bone marrow depression seems most likely to occur when there are leuko-erythroblastic changes but this is not necessarily a contraindication to treatment since improvement may result. No significant fall in haemoglobin levels could be attributed to treatment during the time these patients came under observation.

### DISCUSSION

In this group of patients in whom all other forms of treatment from mastectomy to hypophysectomy had failed to control the tumour, thiotepa has led to significant objective improvement in about one third for over six months at a time when rapid deterioration was anticipated. The prolongation of life and relief of symptoms has sometimes been significant and worthwhile and the unpleasant consequence of treatment minimal. Although the degree of improvement to be expected in these patients is relatively small, the disappearance of obstructive jaundice and control of troublesome metastases has allowed two women who had previously been confined to bed to return to house work. Constant supervision, regular blood counts and individual adjustment of dosage are of course essential, and the improvement is no more than temporary, but the response compares favourably with the effect of analgesics and the usual care given to the dying patient. In any case the use of thiotepa does not exclude the administration of morphia or cocaine as the comfort of the patient demands. Batemen and Larsen (1956) claimed that 80 per cent of their patients with breast cancer were improved even to the extent of recalcification of bone lesions. They emphasised that a slow response was not unusual and that, as with hormones, maintenance treatment was desirable. On the other hand Ultmann *et al.* (1957) found intravenous thiotepa to be effective in the treatment of ovarian carcinoma, but none of the patients with mammary cancer improved. There was a high incidence of haematological depression, 47 per cent of patients developing an abnormal blood picture while 17 per cent had clinical manifestations of bleeding or toxicity. Leonard, Israels and Wilkinson (1956) also encountered a higher incidence of side effects in patients with a variety of lymphomas who were given doses such as 25 mg. per injection. It seems probable that the use of relatively small doses given intramuscularly for as long as possible is less toxic, more effective and to be preferred to more intensive treatment intravenously. Although the value of triethylene thiophosphoramide is limited, a good response in some of these hypophysectomised women with advanced carcinoma of the breast suggests that such treatment should be tried in certain patients when other more effective remedies have failed.

### SUMMARY

Eighteen women with advanced metastatic carcinoma of the breast have been treated with intramuscular triethylene thiophosphoramide in doses of up to a total of 560 mg. for periods of up to 56 weeks. Objective improvement was noted in 10 patients (56 per cent), in seven of whom improvement was maintained for eight weeks or more with a maximum of 56 weeks. It is considered that prolonged intramuscular treatment with thiotepa is worthwhile in patients who have failed to respond to, or relapsed after, hormone therapy or endocrine surgery.

I am indebted to Mr. E. J. Radley Smith, Dr. W. J. D. Fleming and Dr. D. N. Baron for their kind co-operation, and to the British Empire Cancer Campaign for their generous financial assistance.

### REFERENCES

BARON, D. N., GURLING, K. J. AND RADLEY SMITH, E. J.—(1958) *Brit. J. Surg.*, **45**, 593.
BATEMAN, J. C.—(1955) *New Engl. J. Med.*, **252**, 879.
*Idem* AND LARSEN, N. J.—(1956) *J. Amer. geriat. Soc.*, **4**, 341.
HUGGINS, C. and McCARTHY, J. D.—(1957) *Cancer Res.*, **17**, 1028—32.
LEONARD, B. J., ISRAELS, M. C. G. AND WILKINSON, J. F.—(1956) *Lancet* ii, 1017.
LUFT, R., OLIVECRONA, H., IKKOS, D., NILSSON, L. B. AND MOSSBERG, H.—(1958) in ' Endocrine Aspects of Breast Cancer ', Ed. Currie, A. R. Edinburgh (E. & S. Livingstone), p. 27.
RAY, B. S. AND PEARSON, O. H.—(1956) *Ann. Surg.*, **144**, 394.
SHAY, H. AND SUN, D. H.—(1955) *Cancer*, **8**, 498.
*Idem*, ZARAFONETIS, C., SMITH, N., WOLDOW, I. and SUN, D. H.—(1953) *Arch. intern. Med.*, **92**, 628.
ULTMANN, J. E., HYMAN, G. A., CRANDALL, C., NAUJOKS, H. AND GELLHORN, A.—(1957) *Cancer*, **10**, 902.